# Preventing measles transmission in ambulatory pediatric settings during peak respiratory viral season

**DOI:** 10.1017/ash.2023.401

**Published:** 2023-09-29

**Authors:** Katia Halabi, Tammie Hull, Randi Pfeffer, Payal Patel, Sarah King, Randal De Souza, Matthew Washam

## Abstract

**Background:** Measles is a highly transmissible respiratory virus that presents with nonspecific prodromal symptoms followed by a characteristic cephalocaudal rash. In the prodromal phase, children with measles can be challenging to differentiate from children with other circulating respiratory viral infections. A measles outbreak in Central Ohio starting in October 2022 coincided with a national surge in children with viral respiratory infections which presented unique challenges in preventing healthcare transmission in the pediatric ambulatory setting. **Methods:** Following initial identification of presumed community transmission of measles in Central Ohio in November 2022, a multidisciplinary measles response team was convened at Nationwide Children’s Hospital (NCH) to prevent secondary healthcare transmission via rapid-cycle quality improvement. Prevention efforts were focused broadly across NCH ambulatory locations in Central Ohio, including the main campus and offsite emergency departments, regional urgent cares, and primary care network. Preliminary risk factors were identified via chart review of initial cases, which included vaccine status, ZIP code of residence, and known daycare or household exposure. These risk factors were used to guide an intervention bundle comprising enhanced screening at registration and triage, creation of electronic medical record alerts to identify at-risk patients, increased clinician education, and expanded community messaging. As the outbreak evolved, risk factors were updated, and interventions were adjusted to adapt response. Outcome metrics included total patient exposures as well as the relative exposure score. The exposure score was an internal metric derived using the vaccine status of exposed patients and ventilation at the site of exposure to assess likelihood of secondary cases occurring from an exposure. **Results:** In total, 65 patients with measles were seen at NCH facilities between October 29 and December 8, 2022. The outbreak response was divided into 4 periods: (1) cases identified retrospectively prior to first diagnosis (October 29–November 7, 2022), (2) initial case discovery (November 8–14, 2022), (3) implementation of prevention bundle (November 15–28, 2022), and (4) updates to the response (November 29–December 8, 2022). Ambulatory healthcare exposures and incidence of secondary cases decreased over the outbreak periods in response to implementation of the prevention bundle (Fig. and Table). **Conclusions:** An outbreak of measles occurring simultaneously with peak respiratory viral season presented challenges in early identification of suspected cases and mitigation of healthcare exposure. Transmission was effectively prevented following rapid deployment of a prevention bundle adjusted in real-time through rapid-cycle quality improvement. Ongoing longitudinal vaccination efforts are needed to sufficiently mitigate transmission risk in communities with under-vaccinated populations.

**Figure:**
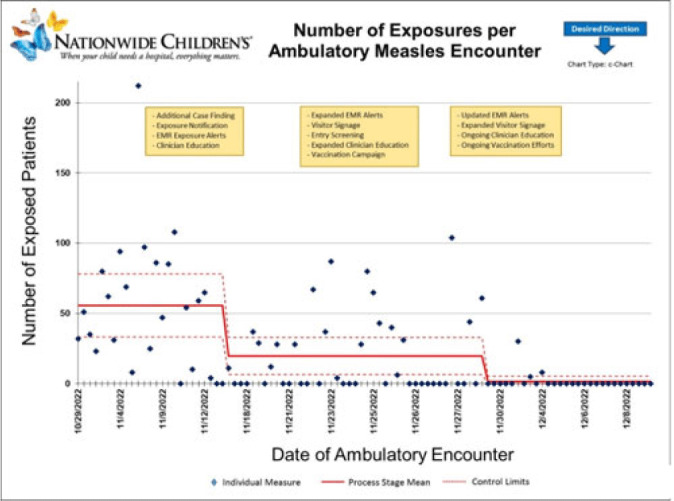

**Figure:**
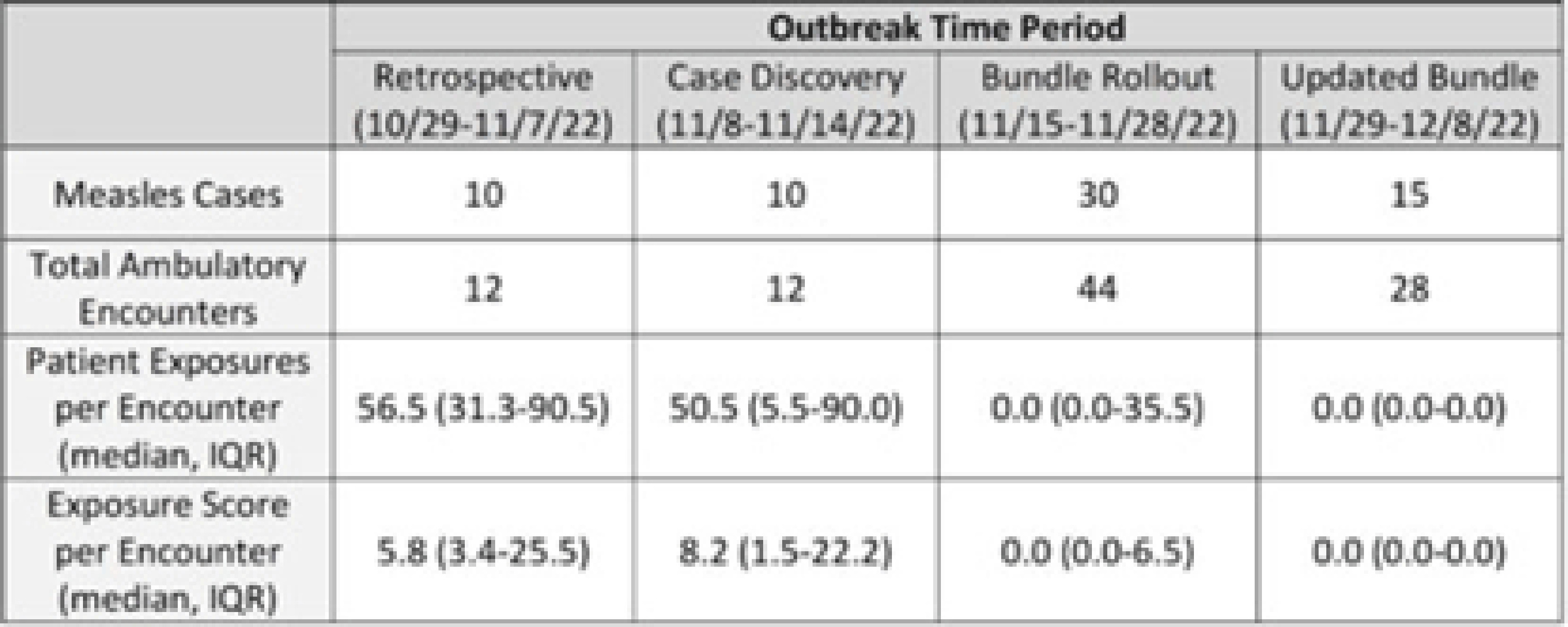

**Disclosures:** None

